# Epithelial-Myoepithelial Carcinoma: Insights From a Case Report

**DOI:** 10.7759/cureus.69544

**Published:** 2024-09-16

**Authors:** Preethi N Sharma, Alka Hande, Swati K Patil, Archana M Sonone, Aayushi Pakhale, Padmashri P Kalmegh, Samiha Khan

**Affiliations:** 1 Oral and Maxillofacial Pathology, Sharad Pawar Dental College and Hospital, Datta Meghe Institute of Higher Education and Research, Wardha, IND; 2 Oral Pathology and Microbiology, Sharad Pawar Dental College and Hospital, Datta Meghe Institute of Higher Education and Research, Wardha, IND

**Keywords:** alpha smooth muscle actin, biphasic, dual population, ductal, epithelial myoepithelial carcinoma, immunohistochemistry, maxillary sinus, p63, s100, salivary gland neoplasm

## Abstract

Epithelial-myoepithelial carcinoma (EMC) is an uncommon malignancy that usually originates in the salivary glands. Its occurrence in the maxillary sinus is rare. We present a case of a 68-year-old male patient who has been experiencing swelling in the upper right back region of the jaw for the past three months. A computerized tomography scan of the paranasal sinus suggested the possibility of a malignant neoplastic mass originating from the right maxillary sinus, with significant local extension and bony erosions. Histopathology examination of EMC revealed a tubular morphology lined with inner luminal cells and outer clear cells of myoepithelial origin. For confirmatory diagnosis, immunohistochemistry (IHC) markers were applied, in which the inner lining of cells was positive for S100, while the outer lining of cells tested positive for both p63 and S100. The patient underwent a hemi-maxillectomy of the right side under general anesthesia.

## Introduction

Epithelial-myoepithelial carcinoma (EMC) is a rare salivary gland tumor, accounting for less than 1% of all neoplasms in these glands [1]. It occurs more frequently in females, particularly in their seventh decade [2]. In 1991, EMC was recognized as a distinct entity by the World Health Organization [3]. The primary site involved is the parotid gland; however, maxillary sinus involvement is rare [4]. There are exceptionally rare instances documented in extraoral areas such as the larynx [5], primitive skin [6], and nasal septum [7]. It is generally studied as a low-grade neoplasm with a slight propensity for nodal and hematogenous spread, but a higher likelihood of regional recurrence [8]. It may occur de novo or as a progression from a benign salivary gland neoplasm [9-11]. The pathophysiology is presumed to be of intercalated duct origin. Clinical presentations of EMC are broad and vary with the location. The differential diagnosis includes salivary gland tumors showing myoepithelial differentiation with or without ductal formation, and those consisting of clear cells, such as pleomorphic adenoma, myoepithelioma, myoepithelial carcinoma, a clear cell variant of mucoepidermoid carcinoma, and adenoid cystic carcinoma. Histopathologically, it exhibits a dual collection of cells, comprising clear cells of myoepithelial origin and ductal-lined epithelial cells. The characteristics of EMC include the growth pattern, sharp discrimination from the hypocellular hyalinized stroma, and the retraction artifact between the ductal and myoepithelial cells. These histologic findings form distinctive areas when EMC arises in a pleomorphic adenoma and help to differentiate EMC from a barely cellular pleomorphic adenoma. Similarly, the absence of a ductal-lined epithelial cell feature distinguishes myoepithelioma and myoepithelial carcinoma from EMC. Imaging modalities, like computed tomography and magnetic resonance, are non-specific, making histopathological and immunohistochemical (IHC) analyses crucial.

The diagnosis remains challenging due to its rarity and the possibility of resemblance to other salivary gland tumors. Advancements in imaging and molecular profiling may help in early diagnosis and targeted interventions. The surgical treatment involves excision with wide margins. Neck dissection, along with lymphadenectomy, is advised only if there is evidence of nodal involvement [12]. Considering the challenging anatomopathological diagnosis and the uniqueness of EMC arising from the maxillary sinus, we present a unique case of EMC involving the maxillary sinus in a 68-year-old male.

## Case presentation

**Figure 1:**
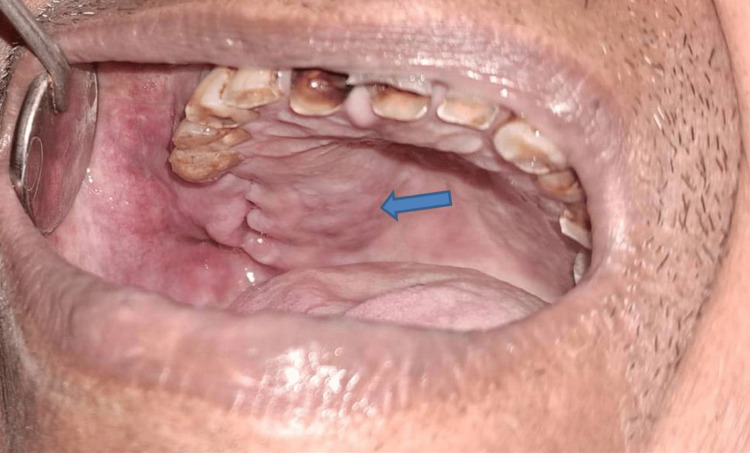
Intraoral lesion with polypoid outgrowth The blue arrow shows the lesion

A 68-year-old male reported the primary concern of discomfort and swelling in the upper right posterior jaw region for the past three months. On examination, intraorally, the swelling was approximately 3 x 2 cm, extending anteroposteriorly from the mesial of the 15 to 18 regions, and superoinferiorly from the maxillary right vestibular region to the alveolar region on the right side. The lesion had a purplish color with a polypoid outgrowth (Figure 1).

On palpation, tenderness was present, induration was also noted, and the consistency was firm, with the lesion fixed to the underlying structure. A contrast-enhanced computerized tomography scan of the paranasal sinus revealed the possibility of a malignant neoplastic mass arising from the right maxillary sinus, with significant local extension and bony erosions.

**Figure 2:**
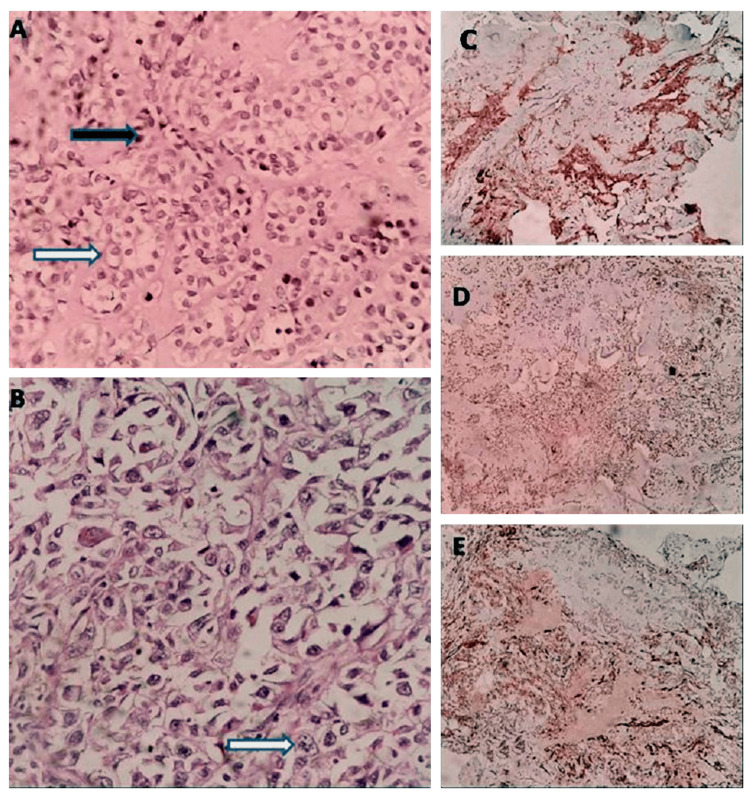
(A) Hematoxylin and eosin (H&E; 4x) staining of the tumor shows the presence of both epithelial (black arrow) and myoepithelial components (white arrow); (B) Mitotic figures (10x; white arrow); (C) Immunohistochemistry (IHC) staining: S100 positive; (D) IHC staining: p63 positive; (E) IHC staining: alpha smooth muscle actin positive

On histological examination, the tumor showed numerous small round to elongated slit-like glandular spaces lined by cuboidal to low columnar luminal cells, and an overlying layer of clear and spindle-shaped myoepithelial cells (Figure 2A). These glands are separated by large sheets of cells with clear cell morphology alternating with areas of plasmacytoid cells. Nuclear morphology is bland. Significant tumor necrosis and mitotic activity are also noted (Figure 2B). For confirmatory diagnosis, IHC markers were applied in which an inner lining of cells was positive for S100 (Figure 2C), and an outer lining of cells, tested positive for p63 (Figure 2D), S100 and alpha smooth muscle actin (Figure 2E).

## Discussion

EMC is a rare neoplasm, mostly affecting the salivary parotid gland. There are case reports published in the literature involving the lacrimal gland [13], auditory meatus [14], floor of the mouth [15], hard palate [16], larynx [17], nasopharynx [18], base of the tongue [19], and buccal mucosa [20]. First described in 1972, EMC is histologically distinguished by the presence of a biphasic nature, comprising myoepithelial cells with clear-staining, surrounding ductal cells lined with epithelium [21,22]. The aggressiveness and prognosis hinge on several factors, including solid tumor growth, nuclear atypia, cytological grading, DNA aneuploidy, necrotic areas, lymphovascular invasion, and cell proliferation indicated by Ki67 [23]. The tumor shows positivity to IHC markers such as S-100 protein, p63, alpha-smooth muscle actin, and cytokeratins [24]. In this case, the neoplasm exhibited a biphasic tubular morphology, with inner luminal cells positive for S100 and outer clear cells of myoepithelial origin positive for p63, S100, and alpha-smooth muscle actin.

Systemic spread and regional lymph node involvement are uncommon, comprising less than 5% of cases [25]. The standard of care is surgical resection with wide margins, which is particularly challenging when EMC arises from the maxillary sinus and extends into the jaw, necessitating multidisciplinary evaluation to preserve speech and swallowing functions [26].

Maxillary sinus EMC is rare, with limited documented cases in the literature. Macroscopically, EMC often presents as multinodular, with well-defined borders and sometimes a capsule [27]. Histologically, EMC has a biphasic cell arrangement in a multinodular pattern. It is made up of outer, clear-staining, polygonal-shaped myoepithelial cells and inner ductal cells lined with epithelium. Many variants exist, with possible solid growth and necrosis, including oncocytic, spindled, clear, and sebaceous types. Vascular invasion is uncommon, while perineural invasion occurs frequently [28]. IHC typically reveals positivity for cytokeratins in the epithelial component, while myoepithelial markers, such as p63, smooth muscle actin, and calponin, are positive in the abluminal counterpart, with S100 typically positive in both components [29].

## Conclusions

EMC is a low-grade tumor demonstrating a dual cell pattern in the histopathological sections. The EMC diagnosis remains difficult, since the tumor may mimic other salivary gland tumors. Therefore, IHC staining can confirm the diagnosis, aiding in determining the best treatment protocol. In this case, the IHC expression of a dual cell population, such as the ductal-lined epithelial cells and the myoepithelial cells, gave a confirmatory diagnosis of EMC. Newer diagnostic methods, such as genomic analysis, chemotherapy regimens, and immunotherapies, might offer better outcomes for managing this rare tumor.
